# Increased EBV infection and relapse following haploidentical hematopoietic cell transplantation in the era of letermovir for cytomegalovirus prophylaxis: a propensity score matching analysis

**DOI:** 10.3389/fcimb.2025.1639463

**Published:** 2025-10-06

**Authors:** Yifei Huang, Shanyu Zhang, Zhiping Fan, Fen Huang, Na Xu, Hua Jin, Min Dai, Li Xuan, Hui Liu, Zhixiang Wang, Jing Sun, Qifa Liu, Ren Lin

**Affiliations:** Department of Hematology, Nanfang Hospital, Southern Medical University, Clinical Medical Research Center of Hematological Diseases of Guangdong Province, Guangzhou, China

**Keywords:** haploidentical donor hematopoietic cell transplantation, letermovir prophylaxis, Epstein-Barr virus infection, relapse, immune reconstitution and function

## Abstract

**Background:**

Letermovir (LTV) is an effective strategy for cytomegalovirus (CMV) reactivation prophylaxis and is increasingly used for allogeneic hematopoietic stem cell transplantation. However, it carries the risk of delayed immune reconstitution. This retrospective study assessed the impact of primary LTV prophylaxis on viral infections, disease relapse, and immune reconstitution in haploidentical hematopoietic stem cell transplantation (haplo-HSCT) recipients.

**Methods:**

Among 462 patients from Nanfang Hospital, propensity score matching created two cohorts: 106 with LTV prophylaxis and 212 without LTV prophylaxis. EBV/CMV infection, relapse, and survival were analyzed by competing risk models and Cox regression. Immune reconstitution and function were assessed by flow cytometry.

**Results:**

LTV prophylaxis had protective effects against CMV viremia, with a 1-year incidence of 32.1% in the LTV group compared with 46.2% in the non-LTV group (P = 0.009). However, the 1-year cumulative incidence of EBV viremia was significantly higher in the LTV group than in the non-LTV group (38.7% vs.13.7%, P<0.001). On multivariate analysis, LTV prophylaxis was a protective factor for CMV viremia (HR = 0.54, P = 0.014) but a risk factor for EBV viremia (HR = 2.69, P<0.001). Additionally, the 1-year cumulative incidence of relapse post-HSCT was notably higher in the LTV group than in the non-LTV group (13.2% vs. 6.1%, P = 0.032). In multivariate analysis, LTV prophylaxis was an independent risk factor for relapse (HR = 2.56, P = 0.024). Lymphocyte subset counts and functions post-transplantation were significantly lower in the LTV group than in the non-LTV group.

**Conclusion:**

LTV prophylaxis might play a dual role in haplo-HSCT recipients, reducing CMV infection but increasing EBV infection and relapse.

## Introduction

Haploidentical hematopoietic stem cell transplantation (haplo-HSCT) is widely used to treat hematologic malignancies and achieves similar outcomes compared with human leukocyte antigen (HLA)-matched sibling donor stem cell transplantation (Guo et al., 2021; Zheng and Tian, 2021). However, the strategies for graft-versus-host disease (GVHD) prophylaxis in haplo-HSCT, which mainly include ex vivo and *in vivo* T-cell depletion (TCD) (Mohty et al., 2024; Giardino et al., 2024), are considered to increase the risk of opportunistic infections especially viral reactivations including Epstein–Barr virus (EBV) and cytomegalovirus (CMV) (Ru et al., 2022).

The novel CMV DNA terminase inhibitor-letermovir (LTV) has been shown to be effective for prophylaxis of CMV reactivation in HSCT recipients (Zhang et al., 2024; Marty et al., 2017; Muhsen et al., 2024). LTV has consistently demonstrated efficacy in reducing the risk of clinically significant CMV infection and CMV disease across various transplantation settings, including matched sibling donors, unrelated donors, and haploidentical donors (Vyas et al., 2023). Historically, the incidence of CMV reactivation has reached 75% in haplo-HSCT recipients (Wang et al., 2019; Huang et al., 2022). With the use of LTV, less than 20% of haplo-HSCT recipients suffer CMV reactivation (Ma et al., 2023; Freyer et al., 2022; Lin et al., 2021; Terao et al., 2021).

CMV reactivation after allo-HSCT has been reported to induce long-lasting expansion of memory-like NK cells, CMV-adapted NK cells and circulating Vδ2^negγδ^ T cells, which might benefit immune reconstruction after HSCT (Litjens et al., 2018; Jang et al., 2019). However, delayed immune reconstitution, including CMV-specific immune reconstitution, was observed among patients receiving LTV prophylaxis in several studies (Zamora et al., 2021; Sperotto et al., 2021; Orofino et al., 2023). Therefore, concern has emerged with respect to the effect of less CMV exposure on other infections especially viral infections (Ito et al., 2013; Elmaagacli et al., 2011; Takenaka et al., 2015). Several studies have reported a greater incidence of EBV reactivation in patients who underwent umbilical cord blood transplantation or haplo-HSCT when LTV was used for CMV prophylaxis (Yan et al., 2024; Kong et al., 2024).

Here, we conducted a retrospective propensity score (PS)-matched cohort study to mainly compare the incidence of EBV reactivation and relapse of disease with and without LTV implementation for CMV prophylaxis after haplo-HSCT, and then to evaluate the impact of LTV on immune reconstruction and function post-transplant.

## Methods

### Study design and patients

Prophylactic LTV was implemented at Nanfang Hospital, Southern Medical University from March 2022 for CMV-seropositive allo-HSCT recipients. LTV prophylaxis was started on the second day of neutrophil engraftment and was continued until day 100 with a dosage of 480 mg daily or 240 mg if concurrent cyclosporin A was used. Consecutive CMV-seropositive haplo-HSCT recipients who received LTV primary prophylaxis for CMV prophylaxis from March 2022 to December 2023 were analyzed in this retrospective study as the LTV cohort. For the non-LTV cohort, propensity score matching (PSM) for baseline variables was used and CMV-seropositive haplo-HSCT recipients without LTV between January 2020 and November 2023 were included. This study was approved by the Medical Ethics Committee of Nanfang Hospital.

### Virus monitoring and GVHD prophylaxis

For all the recipients, the CMV and EBV-DNA loads in the blood were measured regularly by real-time quantitative polymerase chain reaction weekly for the first 3 months after transplantation, once every 2 weeks from the 4^th^ to the 9^th^ month post-transplantation and then once per month from the 10^th^ to the 12^th^ month. Once CMV or EBV-DNA in the blood was positive, the viral loads were detected once again the next day. If positive, viral loads were monitored twice a week. The preemptive treatment threshold is defined as either two consecutive positive CMV PCR results within one week or a single result >500 copies/mL. First-line therapies include ganciclovir and valganciclovir, while second-line options consist of foscarnet, cidofovir, immunoglobulin and cytotoxic T lymphocytes (CTLs) (2022;Xuan et al., 2012). Upon initial detection of positive EBV-DNA in blood (>500 copies/mL), repeat viral load testing was performed the following day. For patients with two consecutive positive EBV-DNA results, the EBV preemptive strategy was initiated, including antiviral therapy (ganciclovir, acyclovir, or foscarnet), intravenous immunoglobulin (0.4 g/kg/day × 3 days), or immunosuppression reduction. In cases of persistently positive EBV-DNA across four consecutive tests with an upward trend, weekly rituximab (375 mg/m²) was administered until viral clearance or for a maximum of 4 weeks (Xuan et al., 2012). ATG (ImtixSangstat, Lyon, France) was administered at 2.5 mg/kg/day from days -3 to -1.

### Flow cytometry analysis

T cell and NK cell subset reconstitution was analyzed using flow cytometry at +1, +2, and +3 months after allo-HSCT in the PSM-matched population to evaluate the overall immune reconstitution process. Besides, functional analysis of cellular immunity was performed to assess the potential impact of LTV prophylaxis on immune function.

Peripheral blood mononuclear cells were isolated from fresh anticoagulated blood via Ficoll density gradient centrifugation and assayed via a FACS CANTO II flow cytometer (BD Biosciences). T cell phenotyping was performed using directly conjugated monoclonal antibodies CD3 (PerCP), CD8 (FITC-A), and CD4 (FITC-A). For NK cell surface staining to identify cell subsets, CD3 (PerCP), CD56 (PE-A), and CD16 (FITC) were used. Concurrently with T cell and NK cell phenotyping, monoclonal antibodies PD-1 (BV421), TIM-3 (PE-Cy-7-A), and CTLA-4 (APC) were used to detect T cell and NK cell exhaustion markers.

T cell and NK cell functional assays involved non-specific stimulation with phorbol myristate acetate (PMA, 50 ng/mL) and ionomycin (Ino, 1 μg/mL) to induce the secretion of intracellular cytokines. After permeabilization, intracellular cytokines interferon-γ (IFN-γ, PE-Cy-7-A), tumor necrosis factor-α (TNF-α, APC), granzyme B (APC), and perforin (PE-Cy-7-A) were detected. The acquired data were further analyzed using BD-FACSDiva™ software. The flow cytometric results are presented as the percentage of positive cells (**Additional File: Figures S1, S2**).

### Definitions

CMV and EBV viremia were both defined as the presence of more than 500 copies/mL in the blood twice consecutively (Lin et al., 2019). The diagnosis of EBV- and CMV- associated diseases were according to the guidelines and our previous description (Lin et al., 2019; Ljungman et al., 2002). Late-onset CMV reactivation was defined as CMV reactivation occurred after 100 days after transplantation (Rowe et al., 2013; Liu et al., 2022). Breakthrough CMV infection was defined as an infection occurring during LTV administration (Perchetti et al., 2023). Patients’ COVID-19 status before HSCT is determined by pre-transplant serological antibody testing and nucleic acid testing (Nuccetelli et al., 2020). Engraftment, relapse, non-relapse mortality (NRM), overall survival (OS), and disease-free survival (DFS) were assessed as previously described (Zeng et al., 2021; Hu et al., 2024). GVHD-free and relapse-free survival (GRFS) was defined as survival without the following events: grade III-IV acute GVHD (aGVHD), severe chronic GVHD (cGVHD), disease relapse, or death from any cause after haplo-HSCT (Li et al., 2023). Organ scoring and global assessment of cGVHD were performed according to the 2014 National Institutes of Health consensus criteria (Filipovich et al., 2005), while aGVHD was evaluated based on the criteria established by the Mount Sinai Acute GVHD International Consortium (Schoemans et al., 2018).

### Statistics

PSM for baseline variables (recipient age and sex, disease, graft type, conditioning regimen, human leukocyte antigen, pretransplant remission status, donor age and sex, CMV/EBV serostatus, mononuclear cells graft) used Logistic regression with the nearest-neighbor method. Caliper settings were set to less than 0.2 to restrict the distance between matched units. Matching ratio was 1:2 for each LTV-non-LTV cohort. Postmatching balance was evaluated with the standardized mean difference, and the optimal balance was considered as <0.2. The chi-square test and Fisher’s exact test were used for categorical or hierarchical features, and Wilcoxon rank-sum test was used to compare continuous variables. Relapse, non-relapse mortality and viral infections were evaluated using the Fine-Gray method (Austin and Fine, 2017) (package cmprsk of R), taking into account cumulative incidence. Correlated risks were estimated with Competing risk model (package FGR of R). Competing events were defined as follows: for relapse, death without relapse; for non-relapse mortality, relapse/progression; for viral infection, death without viral infection. The Kaplan-Meier survival curve and Log-rank test are used for OS, DFS and GRFS. A Cox proportional hazards model was used to evaluate the associations of patient and transplant characteristics with outcomes in a multivariate analysis. The level of statistical significance was set at P < 0.05. Statistical analyses were performed using R version 4.3.3 and SPSS 26.0.

## Results

### Patient characteristics

A total of 462 patients were screened in this study including 106 in the LTV group and 356 in the non-LTV group. The clinical characteristics are shown in **Table 1**. Among the overall cohort, the patient and transplant characteristics were comparable between the LTV group and the non-LTV group, except for differences in graft type (P = 0.016). After PSM, 212 of the 356 patients in the non-LTV group were randomized. The patient characteristics were well-balanced with PSM, and there were no significant differences between the groups (**Table 1**; all P > 0.05, SMD < 0.2). In the LTV group, LTV was started at a median of 13 days (range, 9-24) after haplo-HSCT and was administered for a median duration of 84 days (range, 58-103) post-HSCT.

**Table 1:** Patients’ and donors’ baseline characteristics.

Before PSM					After PSM			
Characteristics	LTV (n = 106)	non-LTV (n = 356)	P	SMD	LTV (n = 106)	non-LTV (n = 212)	P	SMD
Recipient age, year			0.263	0.116			0.726	0.018
Median (Min, Max)	41.0 [14.0, 66.0]	38.0 [13.0, 70.0]			41.0 [14.0, 66.0]	39.0 [16.0, 67.0]		
Recipient sex			0.698	0.056			0.839	0.039
Male	66 (62.3%)	212 (59.6%)			66 (62.3%)	128 (60.4%)		
Female	40 (37.7%)	144 (40.4%)			40 (37.7%)	84 (39.6%)		
Disease			0.660	0.177			0.870	0.134
AML	57 (53.8%)	167 (46.9%)			57 (53.8%)	107 (50.5%)		
ALL	28 (26.4%)	104 (29.2%)			28 (26.4%)	57 (26.9%)		
MDS	14 (13.2%)	61 (17.1%)			14 (13.2%)	37 (17.5%)		
CML	1 (0.9%)	7 (2.0%)			1 (0.9%)	2 (0.9%)		
Others	6 (5.7%)	17 (4.8%)			6 (5.7%)	9 (4.2%)		
Graft type			**0.016**	**0.357**			0.391	0.196
PB	2 (1.9%)	12 (3.4%)			2 (1.9%)	5 (2.4%)		
PB+BM	45 (42.5%)	207 (58.1%)			45 (42.5%)	96 (45.3%)		
PB+BM+UCB	11 (10.4%)	21 (5.9%)			11 (10.4%)	11 (5.2%)		
PB+UCB	48 (45.3%)	116 (32.6%)			48 (45.3%)	100 (47.2%)		
Conditioning regimen			0.206	0.199			0.677	0.106
BuCy-based	32 (30.2%)	118 (33.1%)			32 (30.2%)	60 (28.3%)		
TBI-based	19 (17.9%)	86 (24.2%)			19 (17.9%)	47 (22.2%)		
BuFlu-based	55 (51.9%)	152 (42.7%)			55 (51.9%)	105 (49.5%)		
Donor-recipient relationship			0.470	0.094			1.000	0.010
Sibling	34 (32.1%)	130 (36.5%)			34 (32.1%)	69 (32.5%)		
Family	72 (67.9%)	226 (63.5%)			72 (67.9%)	143 (67.5%)		
HLA			1.000	0.005			0.902	0.010
5/10	64 (60.4%)	214 (60.1%)			64 (60.4%)	127 (59.9%)		
>5/10	42 (39.6%)	142 (39.9%)			42 (39.6%)	85 (40.1%)		
Pretransplant remission status			0.990	0.020			1.000	0.015
CR	93 (87.7%)	306 (87.1%)			93 (87.7%)	187 (88.2%)		
Non-CR	13 (12.3%)	46 (12.9%)			13 (12.3%)	25 (11.8%)		
Donor age, year			0.332	0.090			1.000	0.011
Median (Min, Max)	32.5 (9.0, 59.0)	30.0 (9.00, 64.0)			32.5 (9.0, 59.0)	31.0 (9.00, 60.0)		
Donor sex			0.599	0.072			0.964	0.021
Male	78 (73.6%)	273 (76.7%)			78 (73.6%)	154 (72.6%)		
Female	28 (26.4%)	83 (23.3%)			28 (26.4%)	58 (27.4%)		
CMV serostatus			1.000	0.01			0.515	0.097
D+/R+	90 (84.9%)	301 (84.6%)			90 (84.9%)	187 (88.2%)		
D-/R+	16 (15.1%)	55 (15.4%)			16 (15.1%)	25 (11.8%)		
EBV serostatus			0.120	0.226			0.767	0.086
D+/R+	52 (49.1%)	213 (59.8%)			52 (49.1%)	110 (51.9%)		
D-/R+	22 (20.8%)	52 (14.6%)			22 (20.8%)	37 (17.5%)		
D+/R-	32 (30.2%)	91 (25.6%)			32 (30.2%)	65 (30.7%)		
MNC of graft x10^8/kg			0.201	0.204			0.374	0.021
Median (Min, Max)	8.2 (4.7, 11.4)	8.3 (5.1, 18.4)			8.2 (5.1, 13.1)	8.2 (4.7, 12.4)		

PSM, Propensity Score Matching; LTV, Letermovir; AML, Acute Myeloid Leukemia; ALL, Acute Lymphoblastic Leukemia; MDS, Myelodysplastic Syndrome; CML, Chronic Myeloid Leukemia; PB, Peripheral Blood; BM, Bone Marrow; UCB, Umbilical Cord Blood; BuCy, Busulfan and Cyclophosphamide; TBI, Total Body Irradiation; BuFlu, Busulfan and Fludarabine; HLA, Human Leukocyte Antigen; CR, Complete Remission; CMV, Cytomegalovirus; EBV, Epstein-Barr Virus; D, Donor; R, Recipient; MNC, Mononuclear Cells.

The bold value represent P < 0.05.

### CMV viremia and CMV-associated diseases

**Figure 1:**
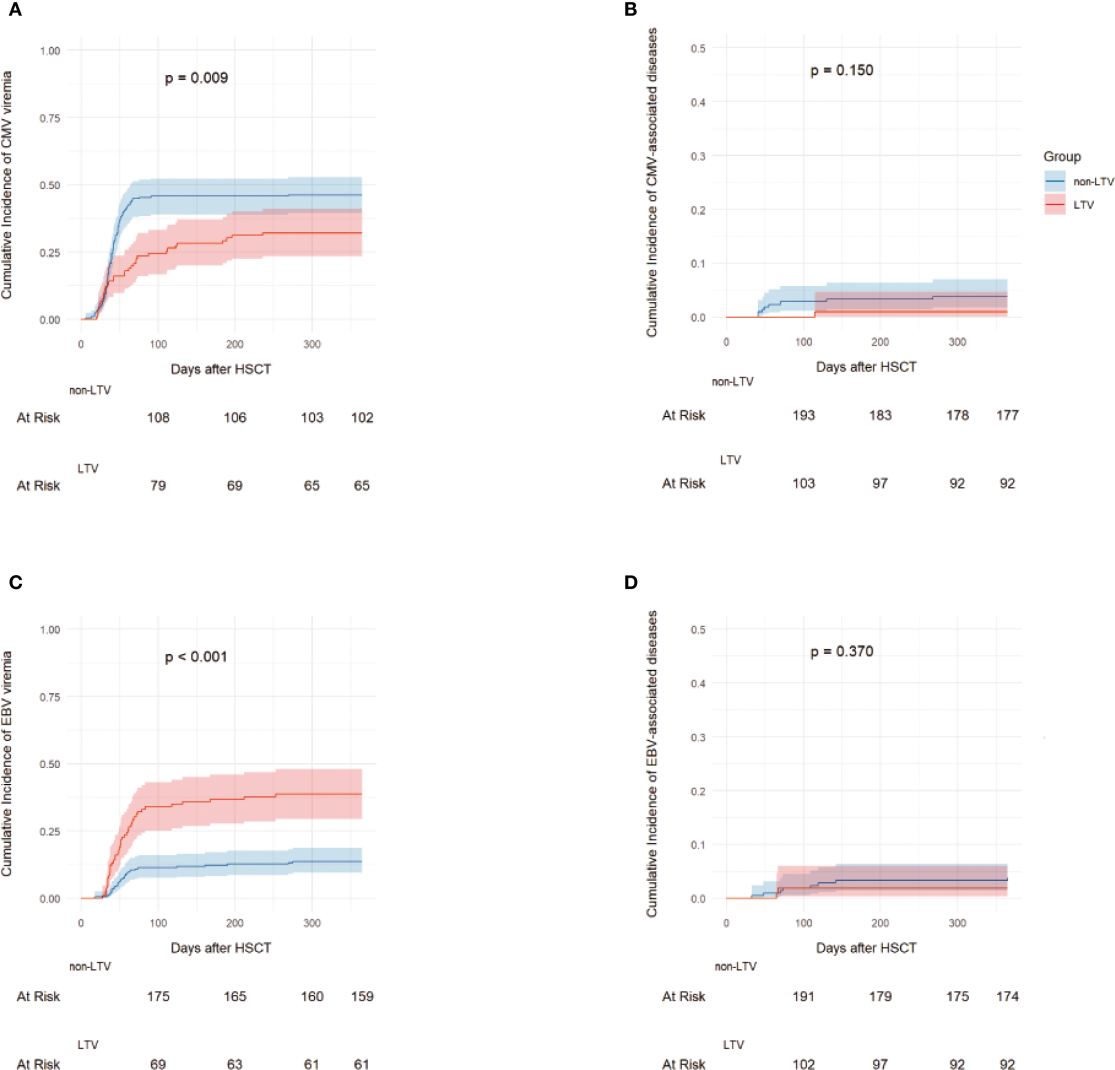
The incidence of CMV and EBV infection. 1-year Cumulative incidences of CMV viremia **(A)**, CMV-associated diseases **(B)**, EBV viremia **(C)** and EBV-associated diseases **(D)** in the LTV and non-LTV groups. LTV, Letermovir; CMV, Cytomegalovirus; EBV, Epstein-barr virus.

The median follow-up was 761.5 days (range, 1–1807) after transplantation. A total of 133 recipients (41.8%) experienced CMV viremia at a median of 40 days (range, 6–366) following haplo-HSCT. The 100-day cumulative incidence of CMV viremia was 24.5% (95% confidence interval [CI], 16.8–33.1%) in the LTV group and 45.8% (95% CI, 38.9–52.3%) in the non-LTV group (P < 0.001). The 1-year cumulative incidence of CMV viremia was significantly lower in the LTV group than in the non-LTV group [32.1% (95% CI, 23.4–41.1%) vs 46.2% (95% CI, 39.4–52.8%); P= 0.009] (**Figure 1A**). Sixteen patients experienced breakthrough CMV viremia at a median of 29.5 days (range, 20-68) after the initiation of LTV prophylaxis. Following first-line treatment, 12 patients (75.0%) achieved CMV-DNA negativity. The remaining 4 patients (25.0%) had persistent CMV infection but successfully cleared the virus after second-line therapy. No patients developed CMV disease or died with sustained CMV-DNA positivity. The 1-year cumulative incidences of late-onset CMV viremia after HSCT in the LTV and non-LTV groups were 8.5% (95% CI, 4.2-14.9%) and 0.5% (95% CI, 0-2.4%), respectively (P <0.001).

During the follow-up period, one patient in the LTV group developed CMV pneumonia at 115 days after haplo-HSCT. In the non-LTV group, eight patients developed CMV-associated diseases including 4 with enteritis, 3 with pneumonia, and 1 with encephalitis (**Figure 1B**). The median time to onset of CMV-associated diseases in the non-LTV group was 52 days (range, 41–268) after haplo-HSCT. The 1-year cumulative incidence of CMV-associated diseases was 0.9% (95% CI: 0.1–4.7%) and 3.8% (95% CI: 1.8–7.0%) in the LTV and non-LTV groups, respectively (P = 0.150). The detailed characteristics of CMV infection are summarized in **Table 2**.

**Table 2:** Features of CMV and EBV infection with and without LTV.

Variable	LTV (n = 106)	non-LTV (n = 212)	P
Time of first CMV viremia, d [Median, (Min, Max)]	56.0 [20.0, 366.0]	40.0 [6.0, 269.0]	0.094
Time of CMV-associated diseases, d [Median, (Min, Max)]	115.0	52.0 [41.0, 268.0]	0.667
Duration of CMV viremia, d [Median, (Min, Max)]	12.0 [4.0, 60.0]	17.0 [2.0, 60.0]	0.255
Highest copies of CMV, copies/ml [Median, (Min, Max)]	2470.0 [650.0, 39300.0]	2825.0 [700.0, 257000.0]	0.746
Time of first EBV viremia, d [Median, (Min, Max)]	51.0 [28.0, 253.0]	52.0 [18.0, 275.0]	0.508
Time of EBV-associated diseases, d [Median, (Min, Max)]	66.0 [65.0, 67.0]	91.0 [33.0, 357.0]	0.400
Duration of EBV viremia, d [Median, (Min, Max)]	14.5 [2.0, 67.0]	17.0 [3.0, 70.0]	0.342
Highest copies of EBV, copies/ml [Median, (Min, Max)]	1610.0 [571.0, 778000.0]	2100.0 [890.0, 38400.0]	**0.043**

LTV, Letermovir; CMV, Cytomegalovirus; EBV, Epstein-Barr Virus.

The bold value represent P < 0.05.

### EBV viremia and EBV-associated diseases

A total of 70 recipients (22.0%) experienced EBV viremia at a median of 51.5 days (range, 18–275) following haplo-HSCT. The 100-day cumulative incidence of EBV viremia was 34.0% (95% CI, 25.1–43.0%) in the LTV group and 11.3% (95% CI, 7.5–16.0%) in the non-LTV group (P < 0.001). The 1-year cumulative incidence of EBV viremia was significantly higher in the LTV group than in the non-LTV group [38.7% (95%CI, 29.4-47.9%) vs. 13.7% (95%CI, 9.5-18.7%), P < 0.001] (**Figure 1C**). In the LTV group, 16 patients with EBV viremia received rituximab preemptive therapy and 1 received EBV-CTL therapy. In the non-LTV group, 15 patients with EBV viremia received rituximab pre-emptive therapy and 6 received EBV-CTL therapy. The overall response rates to therapy were 86.7% and 72.2% for patients with EBV viremia in the LTV and non-LTV groups, respectively (P = 0.413).

During the follow-up period, two patients in the LTV group developed EBV-associated diseases including 1 hemophagocytic lymphohistiocytosis (HLH) and 1 with encephalitis while 8 in the non-LTV group developed EBV-associated diseases including 3 posttransplant lymphoproliferative disorder (PTLD), 3 with enteritis, 1 with encephalitis and 1 with pneumonia. The median time to onset of EBV-associated diseases was 66 days (range, 65-67) in the LTV group and 91 days (range, 33–357) in the non-LTV group. The 1-year cumulative incidence of EBV-associated diseases was 1.9% (95% CI: 0.4–6.1%) and 3.3% (95% CI: 1.5–6.4%) in the LTV and non-LTV groups, respectively (P = 0.370) (**Figure 1D**). Among the 2 patients who developed EBV- associated disease in LTV group, both received rituximab and EBV-CTL therapy but ultimately died due to disease severity. In contrast, although 8 patients experienced EBV- associated disease in non-LTV group, outcomes were comparatively better—only 1 patient with EBV pneumonia died from sepsis, while the remaining cases were controlled or cured. The detailed characteristics of the EBV infection are summarized in **Table 2**.

### Risk factors for CMV and EBV infections

Univariate and multivariate analyses of the risk factors for EBV and CMV infections post-transplantation are shown in **Table 3**. On multivariate analysis, LTV prophylaxis was a protective factor for CMV viremia (HR = 0.54, 95%CI, 0.33-0.88, P = 0.014) but a risk factor for EBV viremia (HR = 2.69, 95%CI, 1.56–4.64, P<0.001). The female donor served as a protective factor for EBV viremia. Grade III-IV aGVHD was identified as a risk factor for CMV viremia, CMV-associated diseases and EBV-associated diseases. Patient sex and age, donor age, conditioning regimen, graft type, HLA typing, patients’ COVID-19 status pre-HSCT and EBV/CMV serostatus did not show any significant influence on the risk of EBV and CMV infections.

**Table 3:** Univariate and multivariate analyses of risk factors for EBV and CMV infections with 1-year after HSCT.

Risk factors	CMV viremia		CMV-associated diseases		EBV viremia		EBV-associated diseases	
	Univariate P (HR,95%CI)	Multivariate P (HR,95%CI)	Univariate P (HR,95%CI)	Multivariate P (HR,95%CI)	Univariate P (HR,95%CI)	Multivariate P (HR,95%CI)	Univariate P (HR,95%CI)	Multivariate P (HR,95%CI)
Group								
LTV vs. non-LTV	**0.010** (0.60, 0.41-0.89)	**0.014** (0.54, 0.33-0.88)	0.180 (0.25, 0.03-1.95)	0.079 (0.16, 0.02-1.23)	**<0.001** (3.33, 2.07-5.34)	**<0.001** (2.69, 1.56-4.64)	0.380 (0.50, 0.11-2.35)	0.619 (0.48, 0.03-8.69)
Patient’s sex								
Female vs. male	0.770 (1.05, 0.75-1.49)	0.915 (1.02., 0.71-1.47)	0.730 (1.26, 0.34-4.68)	0.468 (1.68, 0.41-6.81)	0.170 (0.70, 0.42-1.17)	0.370 (0.78, 0.46-1.33)	0.960 (1.03, 0.29-3.64)	0.918 (1.07, 0.27-4.37)
Patient’s age								
>40 vs. ≤40	0.150 (0.78, 0.56-1.09)	0.271 (0.81, 0.56-1.18)	0.360 (0.52, 0.13-2.08)	0.678 (0.75, 0.20-2.88)	0.480 (1.19, 0.74-1.89)	0.430 (1.26, 0.71-2.21)	0.580 (0.70, 0.20-2.47)	0.974 (1.03, 0.22-4.84)
Donor’s sex								
Female vs. male	0.360 (0.83, 0.56-1.24)	0.427 (0.84, 0.56-1.28)	0.300 (0.33, 0.04-2.64)	0.518 (0.47, 0.05-4.66)	**0.010** (0.42, 0.21-0.81)	**0.023** (0.45, 0.23-0.89)	0.250 (0.30, 0.04-2.32)	0.727 (0.71, 0.10-4.99)
Donor’s age								
>31 vs. ≤31	0.800 (1.04, 0.74-1.47)	0.987 (1.00, 0.71-1.40)	0.750 (0.81, 0.22-2.99)	0.264 (0.48, 0.14-1.73)	0.074 (1.54, 0.96-2.46)	0.160 (1.44, 0.86-2.41)	0.071 (4.14, 0.89-19.4)	0.315 (2.45, 0.43-14.13)
Conditioning regimen								
BU-based vs. TBI-based	0.250 (0.79, 0.53-1.18)	0.661 (0.91, 0.60-1.39)	0.083 (0.31, 0.08-1.16)	0.245 (0.37, 0.07 -2.00)	0.280 (1.42, 0.75-2.71)	0.450 (1.36, 0.61-3.04)	0.420 (2.33, 0.30-18.40)	0.380 (3.69, 0.20-68.39)
Graft type								
UCB vs.non-UCB	0.740 (1.06, 0.75-1.49)	0.876 (1.03, 0.73-1.46)	0.580 (0.69, 0.19-2.57)	0.420 (0.59, 0.17-2.12)	0.350 (1.25, 0.78-2.01)	0.660 (1.12, 0.67-1.88)	0.680 (1.31, 0.37-4.61)	0.776 (1.24, 0.28-5.48)
HLA typing								
5/10 vs. >5/10	0.300 (1.20, 0.85-1.69)	0.169 (1.28, 0.90-1.82)	0.120 (3.03, 0.76-12.10)	0.115 (3.91, 0.72-21.33)	0.380 (0.81, 0.50-1.31)	0.780 (0.93, 0.57-1.52)	0.210 (0.37, 0.08-1.73)	0.436 (0.48, 0.08-3.01)
aGVHD before viral infection								
Grade III-IV vs. Grade0-II	**0.034** (1.79, 1.05-3.07)	**0.036** (1.78, 1.04-3.05)	**<0.001** (10.70, 2.91-34.40)	**0.001** (16.45, 2.93-92.40)	**0.047** (2.03, 1.01-4.07)	0.350 (1.55, 0.62-3.91)	**<0.001** (22.00, 6.37-76.20)	**<0.001** (17.39, 3.40-89.05)
Patients COVID-19 status pre-HSCT								
IgG+ vs. IgG-	0.390 (0.84, 0.55-1.26)	0.439 (1.23, 0.73-2.07)	0 390 (0.40, 0.05-3.17)	0.892 (1.15, 0.15 -9.07)	**<0.001** (2.36, 1.46-3.83)	0.250 (1.40, 0.79-2.50)	0.790 (0.81, 0.17-3.79)	0.853 (1.38, 0.05-40.34)
CMV serostatus								
D+/R+ vs. D−/R+	0.760 (0.92, 0.55-1.56)	0.579 (0.86, 0.50-1.47)	0.410 (1.92, 0.41-9.05)	0.722 (1.39,0.22-8.62)	-	-	-	-
EBV serostatus								
Mismatch vs. match	-	-	-	-	0.270 (1.30, 0.813.-2.07)	0.350 (1.27, 0.77-2.07)	0.940 (1.05, 0.31-3.61)	0.629 (1.43,0.33-6.19)

HR, Hazard ratio; LTV, Letermovir; BU, Busulfan; TBI, Total Body Irradiation; UCB, Umbilical Cord Blood; HLA, Human leukocyte antigen; aGVHD, Acute graft versus host disease; CMV, Cytomegalovirus; EBV, Epstein-barr virus; D, Donor; R, Recipient.

The bold value represent P < 0.05.

### Subgroup analysis

To further examine the influence of LTV prophylaxis on EBV viremia post-transplantation, we performed subgroup analyses stratified by patient sex, patient age, donor sex, donor age, condition regimen, graft type, HLA typing, aGVHD before EBV viremia, patients COVID-19 status pre-HSCT and EBV serostatus. The 1-year incidence of EBV viremia was significantly higher in the LTV cohort than in the non-LTV cohort across all subgroups except for patients with Grade III-IV aGVHD before EBV viremia (**Additional File: Figure S3**).

### GVHD

There were no significant differences in the cumulative incidence of grades I to IV aGVHD between the LTV group and the non-LTV group within 100 days [42.5% (95%CI, 32.9-51.7%) vs. 41.5% (95%CI, 34.8-48.1%), P = 0.780]. The cumulative incidence of grades II to IV acute GVHD for patients with LTV and patients without LTV were 22.8% (95%CI, 15.3-31.2%) and 19.4% (95%CI, 14.4-25.0%), respectively (P = 0.450). No significant differences in grades III and IV acute GVHD were detected between LTV group and non-LTV group [5.7% (95%CI, 2.3-11.2%) vs. 6.6% (95%CI, 4.0-10.5%), P = 0.738]. The cumulative incidence of moderate to severe cGVHD by 1-year post-HSCT was similar between patients with and without LTV, with rates of 17.9% (95%CI, 11.3-25.8%) and 16.5% (95%CI, 11.9-21.8%), respectively (P = 0.730).

### Survival

**Figure 2:**
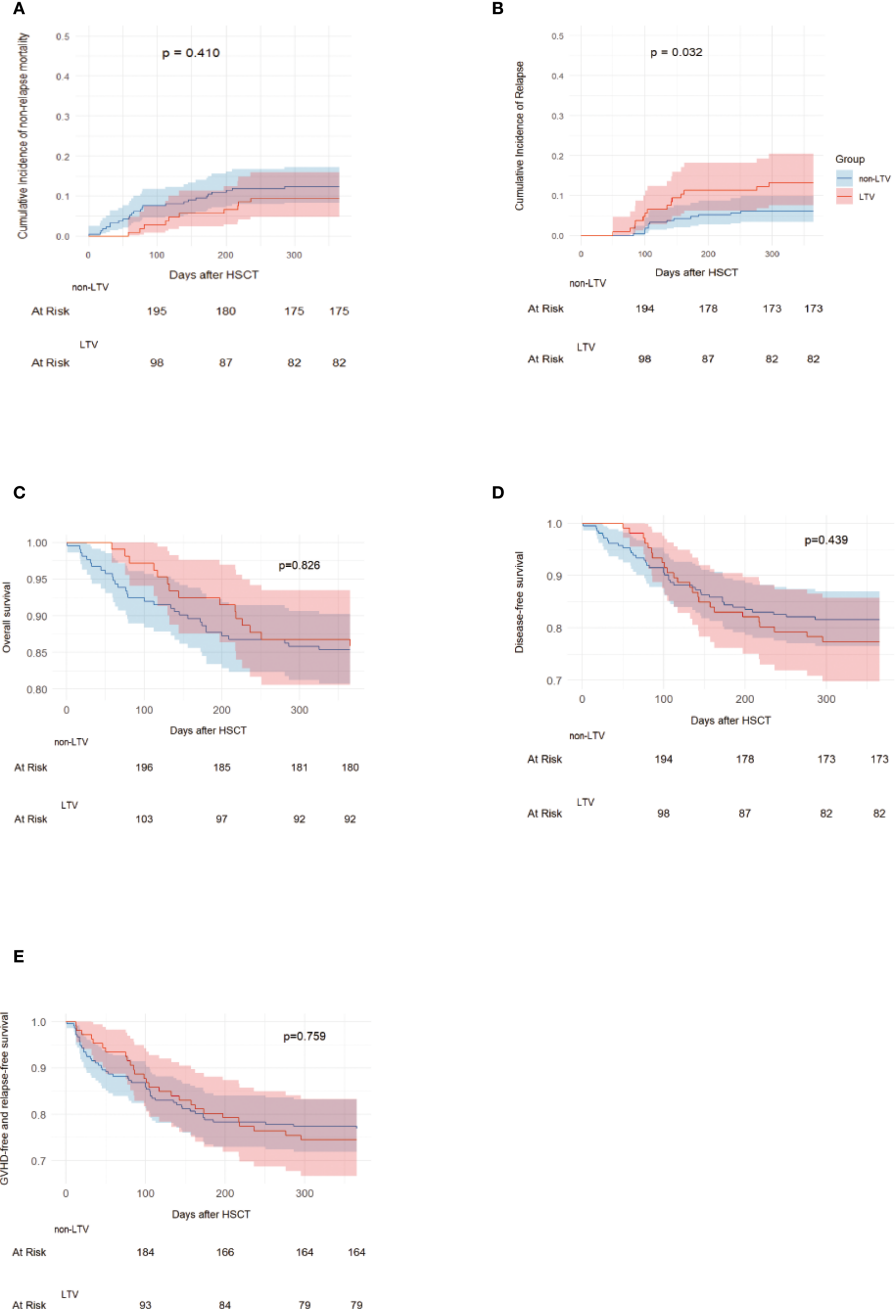
NRM, Relapse, OS, DFS and GRFS in LTV and without LTV groups. Non-relapse mortality **(A)**, Relapse **(B)**, Overall survival **(C)**, Disease-free survival **(D)**, GVHD free, relapse free survival **(E)**. LTV, Letermovir; HSCT, Hematopoietic stem cell transplantation; GVHD, graft versus host disease.

During the follow-up period, 272 patients survived and 49 died, of whom 15 were in the LTV group and 34 were in the non-LTV group. The causes of death are presented in **Additional File: Figure S4**. The 1-year incidence of NRM was 9.4% (95% CI, 4.8–15.9%) in the LTV group and 12.3% (95% CI, 8.3–17.2%) in the non-LTV group (P = 0.410, **Figure 2A**). The 1-year incidence of relapse was 13.2% (95% CI, 7.6–20.4%) in the LTV group and 6.1% (95% CI, 3.4–9.9%) in the non-LTV group (P = 0.032, **Figure 2B**). The 1-year incidence of OS was 85.9% (95% CI, 78.0–91.2%) and 85.4% (95% CI, 80.0–89.5%), DFS was 77.4% (95% CI, 68.5–84.3%) and 82.1% (95% CI, 76.4–86.7%), and GRFS was 74.5% (95% CI, 65.5–81.9%) and 76.9% (95% CI, 70.8–82.1%), respectively, in the LTV and non-LTV groups (OS: P = 0.826, **Figure 2C**; DFS: P = 0.439, **Figure 2D**; GRFS: P = 0.759, **Figure 2E**). In multivariate analysis, LTV prophylaxis was the independent risk factor for relapse (HR, 2.56; 95% CI, 1.13-5.80 P = 0.024). EBV viremia was an independent risk factor for NRM, while Grade III–IV aGVHD was an independent risk factor for NRM, OS, and DFS (**Table 4**; **Supplementary Table S1**).

**Table 4:** Uni- and multivariate fine-gray competing risk regression analyses for NRM and relapse.

Risk factors	NRM		Relapse	
	Univariate P (HR,95%CI)	Multivariate P (HR,95%CI)	Univariate P (HR,95%CI)	Multivariate P (HR,95%CI)
Group				
LTV vs. non-LTV	0.410 (0.74, 0.36-1.52)	0.298 (0.63, 0.26-1.51)	**0.035** (2.25, 1.06-4.77)	**0.024** (2.56, 1.13-5.80)
Patient’s sex				
Female vs. male	0.710 (0.88, 0.45-1.74)	0.775 (0.89, 0.40-1.97)	0.300 (1.49, 0.70-3.16)	0.122 (1.95, 0.84-4.54)
Patient’s age (Median)				
>40 vs.≤40	0.058 (1.93, 0.98-3.80)	0.054 (2.22, 0.98-4.99)	0.710 (1.15, 0.54-2.45)	0.786 (1.13, 0.48-2.66)
Conditioning regimen				
BU-based vs. TBI-based	0.790 (0.90, 0.42-1.96)	0.599 (0.78, 0.31-1.98)	0.820 (0.90, 0.37-2.22)	0.952 (1.04, 0.33 -3.27)
Graft type				
UCB vs. non-UCB	0.900 (0.96, 0.50-1.84)	0.604 (0.83, 0.41-1.68)	0.320 (0.68,0.32-1.45)	0.879 (0.94,0.40-2.21)
Disease status				
CR vs. non-CR	0.710 (0.84, 0.33-2.14)	0.359 (1.76, 0.53-5.91)	0.470 (1.71, 0.40-7.29)	0.233 (3.38, 0.46-24.93)
Cytogenetics				
High-risk vs. low/ Intermediate-risk	0.340 (1.48, 0.66-3.30)	0.449 (1.41, 0.58-3.44)	0.100 (0.19, 0.03-1.40)	0.104 (0.18, 0.02-1.42)
aGVHD				
Grade III-IV vs. Grade0-II	**<0.001** (4.11, 1.87-9.06)	**0.005** (3.76, 1.48-9.56)	0.980 (0.98, 0.23-4.15)	0.691 (1.40, 0.26-7.44)
CMV viremia				
Y vs. N	**0.006** (2.59, 1.31-5.12)	0.105 (1.87, 0.88-3.98)	0.370 (0.70, 0.31-1.54)	0.985 (0.99, 0.37-2.63)
EBV viremia				
Y vs. N	**0.004** (2.63, 1.36-5.06)	**0.047** (2.22, 1.01-4.88)	0.330 (1.51, 0.66-3.41)	0.493 (1.37, 0.57-3.34)
CMV serostatus				
D+/R+ vs. D−/R+	0.180 (0.38, 0.09-1.58)	0.075 (0.31, 0.09-1.12)	0.130 (1.99, 0.81-4.87)	0.126 (2.23, 0.80-6.25)

HR, Hazard ratio; LTV, Letermovir; BU, Busulfan; TBI, Total Body Irradiation; UCB, Umbilical Cord Blood; CR, Complete Remission; aGVHD, acute graft versus host disease; CMV, Cytomegalovirus; EBV, Epstein-barr virus; Y, Yes; N, No; D, Donor; R, Recipient.

The bold value represent P < 0.05.

### Immune reconstitution and function

**Figure 3:**
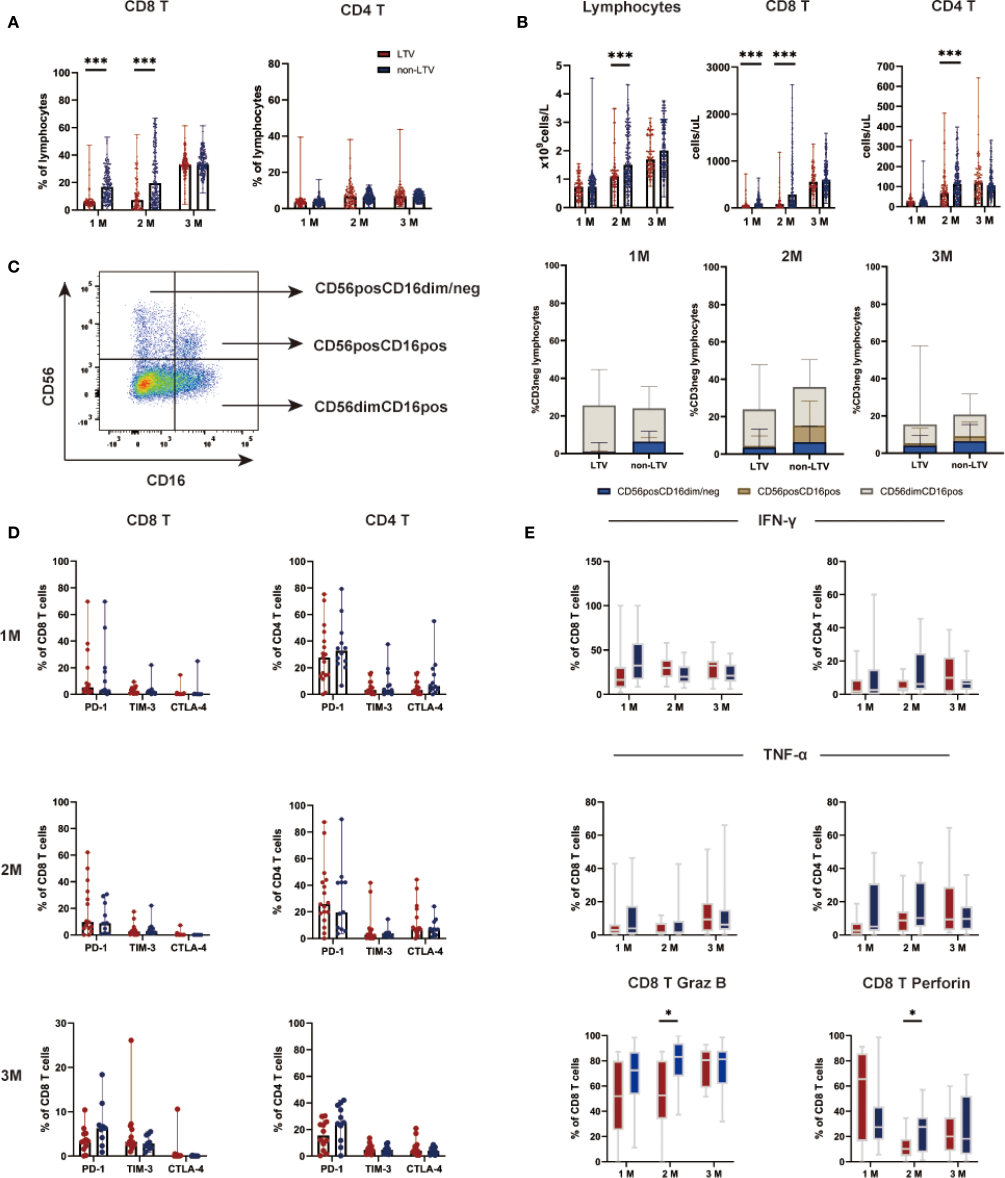
Lymphocyte subsets and functions within three months post-transplantation. CD8 and CD4 T lymphocyte subsets percentages **(A)**, CD8 and CD4 lymphocyte subsets counts **(B)**, NK lymphocyte subsets percentages **(C)**, Expression of exhaustion markers on lymphocyte subsets **(D)**, Function of lymphocyte subsets **(E)** within three months post-transplantation in the LTV and non-LTV groups. LTV, Letermovir; M, Month; *P < 0.05, ***P < 0.001.

Immune reconstitution was analyzed in 272 patients who had continuous and complete immune reconstitution data including 95 in the LTV group and 177 in the non-LTV group (**Figure 3** and **Additional File: Table S2**). At 1 and 2 months post-transplant, the median percentage of CD8 T cells was significantly lower in the LTV group compared with the non-LTV group (both P < 0.001, **Figure 3A**). No significant difference was observed in the median percentage of CD4 T cells and NK cells between the two groups at 1, 2, 3 months post-transplant. (**Figure 3A**; C). At 2 month post-transplant, the median counts of lymphocytes were significantly lower in the LTV group compared to the non-LTV group (P < 0.001, **Figure 3B**). At 1 and 2 months post-transplant, the median counts of CD8 T cells were significantly lower in the LTV group compared to the non-LTV group (both P < 0.001, **Figure 3B**). At 2 month post-transplant, the median counts of CD4 T cells were significantly lower in the LTV group compared to the non-LTV group (P < 0.001, **Figure 3B**).

Further analysis of T cell function was performed in 29 patients including 17 LTV group and 12 in non-LTV group. The results revealed that, at 2 months post-transplant, the Granzyme B (P = 0.014; **Figure 3E**) and perforin (P = 0.040; **Figure 3E**] expression in CD8 T cells in the LTV group was significantly lower than in the non-LTV group. The expression of PD-1, TIM-3, and CTLA-4 on CD8 and CD4 T cell subsets were similar between the two groups at 1, 2, 3 months post-transplant (**Figure 3D**).

Among patients with immune reconstitution cell count analysis, 51 (18.75%) developed EBV reactivation (EBV+ group); while among patients with immune function assessment, 16 (55.17%) experienced reactivation. Comparative analysis revealed that 1 month post-transplant, the EBV+ group exhibited significantly lower absolute counts of lymphocytes (P <0.001)), CD8 T cells (P <0.001), and CD4 T cells (P = 0.037) compared to the EBV- group, with CD8 T cell counts remaining significantly lower at 2 month post-transplant (P <0.001). Regarding immune function, the EBV+ group showed markedly reduced expression of granzyme B (P = 0.013) and perforin (P = 0.028) by CD8^+^ T cells at month 1 post-transplant relative to the EBV- group (**Supplementary Figure S5**).

## Discussion

In the current study, LTV prophylaxis, although reducing the risk for CMV viremia, was associated with a significantly increased incidence of EBV viremia and primary disease relapse. Besides, our results demonstrated that LTV may delay T-lymphocyte reconstitution and impair its function, which could potentially contribute to these outcomes.

haplo-HSCT has become widely adopted worldwide, particularly through T-cell-replete strategies, such as those involving post-transplantation cyclophosphamide (PTCy) or ATG-based protocols. The ATG-based regimen is one of the most commonly used GVHD prophylaxis strategies for haplo-HSCT in China but is associated with a relatively high risk of CMV and EBV infection after HSCT (Wang et al., 2023; Ru et al., 2020; Yang et al., 2019). LTV has now been recommended for preventing CMV infection in CMV-seropositive recipients (Kong et al., 2024; Cesaro et al., 2023; Febres-Aldana et al., 2024). In several retrospective and observational studies on the efficacy of LTV, which included patients who underwent haplo-HSCT with ATG prophylaxis, the results showed that the incidence of post-transplant CMV viremia decreased to 20%-35% following LTV prophylaxis (Toya et al., 2024; Hopff et al., 2024). In addition, we found that the incidence of late-onset CMV and breakthrough CMV infections was also consistent with those in previous studies (Włodarczyk et al., 2024; Khawaja et al., 2023).

Several studies have explored the impact of LTV prophylaxis on other members of the herpesvirus family, such as HHV-6 (Kampouri et al., 2023; Terao et al., 2024) and EBV (Yan et al., 2024; Kong et al., 2024; Pei et al., 2024). Up to now, research has not revealed that using LTV increases the risk of HHV-6 reactivation or HHV-6 encephalitis (Kampouri et al., 2023; Terao et al., 2024). Studies have shown that the significant increase in EBV infection following LTV prophylaxis is primarily observed in allo-HSCT patients including both adult and pediatric populations (Zhen et al., 2025; Oikonomopoulou et al., 2025). A real-world experience demonstrated that EBV reactivation was more frequent in patients receiving umbilical cord blood transplantation with LTV prophylaxis (Yan et al., 2024). Kong et al. conducted a study involving 230 patients received haplo-HSCT, and the results showed that the incidence of EBV reactivation after HSCT was significantly higher in the patients who received LTV prophylaxis than in those who did not (Kong et al., 2024). Furthermore, a recent study indicated that haplo-HSCT recipients receiving LTV had higher risk of PTLD compared to those who did not receive LTV (Pei et al., 2024). In our study, a higher incidence of EBV viremia in the LTV group was found compared to the non-LTV group. Multivariate analysis showed that LTV prophylaxis was the only risk factor for EBV reactivation after haplo-HSCT. Subgroup evaluations further confirmed that the use of LTV increased the incidence of EBV viremia in all subgroups, except for the grade III-IV aGVHD subgroup.

Previous studies have shown that LTV prophylaxis might be associated with a delay in polyfunctional CMV-specific cellular immune reconstitution (Zamora et al., 2021; Giménez et al., 2023). LTV prophylaxis was associated with delayed polyfunctional CMV-specific T-cell subsets reconstitution and decreased CMV antigens responses at 3 months after HSCT compared with preemptive antiviral therapy (Zamora et al., 2021). Sperotto et al. (2021) conducted a study involving 110 HSCT patients with 55 receiving preemptive antiviral treatment and 55 receiving LTV, and the results showed that the LTV group experienced impaired recovery of CD4 and CD8 T cells at days +60 and +90 after HSCT. A recent study including allo-HSCT patients aged 14 years and older, of whom 96.5% received ATG as GVHD prophylaxis followed by LTV, found that compared to non-LTV patients, the incidence of EBV viremia was significantly higher at 200 days post-transplant. Additionally, the LTV group showed a reduction in lymphocytes and CD8 T cells after transplantation (Zhen et al., 2025). Consistent with these findings, our study also observed that patients in the LTV group exhibited lower counts of total lymphocytes, CD4, and CD8 T cells compared to those who did not receive LTV. Furthermore, in our study, patients receiving LTV prophylaxis had reduced Granzyme B and perforin secretion by CD8 T cells at +2 months post-transplant compared to those not receiving LTV. These results suggests that LTV may lead to delayed immune reconstitution and impaired lymphocyte function. CMV reactivation was considered to drive posttransplant T-cell reconstitution (Scheper et al., 2013; Schäfer et al., 2024). While our study did not directly assess CMV-CTL subset reconstitution, CMV-CTLs are a critical component of CD8 T cells, and their quantitative and functional recovery may contribute to overall cellular immune reconstitution. we also conducted an exploratory analysis comparing immune reconstitution and function between patients with and without EBV reactivation. The results demonstrated that patients experiencing EBV reactivation exhibited impaired lymphocytes, CD8 and CD4 T cells reconstitution and CD8 T cells functional deficits. Therefore, we presumed that the impairment of immune reconstitution and function due to the reduce of exposure of CMV following LTV prophylaxis may be attribute to increased EBV reactivation.

In our study, despite a higher incidence of EBV viremia in the LTV group, the median copy peak of EBV viremia was lower than that in non-LTV group, and LTV did not significantly affect the incidence of EBV disease. This is consistent with findings from Kong’s study (Kong et al., 2024) which reported that the proportion of patients in the LTV group with low EBV-DNA loads (≥ 5 × 10² to < 1 × 10^4^ copies/mL) was significantly higher than that in the control group. This may be attributed to early-stage pre-emptive therapy.

Interestingly, we observed a significantly higher relapse rate in the LTV group compared to the non-LTV group, and LTV prophylaxis was identified as an independent risk factor for relapse in this study. Research from Japan also found that the use of LTV prophylaxis was associated with an increased risk of relapse after transplantation (Akahoshi et al., 2022). In 1986, Swedish research found that patients with CMV infection after HSCT had a lower relapse rate than those without the infection (Lönnqvist et al., 1986). Subsequent studies confirmed that CMV reactivation protects against relapse in acute leukemia (Elmaagacli et al., 2011; Manjappa et al., 2014), with a major Japanese study of 3,539 allo-HSCT patients demonstrating that post-transplant CMV reactivation is an independent protective factor for relapse (Takenaka et al., 2015). Recent findings suggest that CD57+/CD27- CD4+ cells expanded during CMV exposure may eliminate CMV-infected leukemic cells (Yeh et al., 2021), potentially explaining the increased relapse rate following LTV treatment.

This study has certain limitations. First, although the cohorts were well matched, other variables (such as the use of corticosteroids and other immunosuppressive agents) may influence immune reconstitution and the probability of EBV reactivation after HSCT. Additionally, the immune reconstitution of EBV-CTL, which might better reflect the immune status for EBV, in the two groups also needs further study. LTV primary prophylaxis may exert a dual effect in ATG-based haplo-HSCT recipients—reducing CMV infection while increasing the risk of EBV infection and disease relapse. This phenomenon may be attributed to the impact of LTV on post-transplant lymphocyte reconstitution and function. These findings suggest the need for further investigation into virus-specific immune reconstitution following LTV administration and highlight the potential necessity for personalized prophylactic and monitoring strategies.

## Data Availability

The original contributions presented in the study are included in the article/**Supplementary Material**. Further inquiries can be directed to the corresponding authors.
