# Supplementary material for: Increased EBV infection and relapse following haploidentical hematopoietic cell transplantation in the era of letermovir for cytomegalovirus prophylaxis: a propensity score matching analysis
Source: Front Cell Infect Microbiol. 2025 Oct 6;15:1639463. doi: 10.3389/fcimb.2025.1639463 (PMC12535987; doi:10.3389/fcimb.2025.1639463)
Supplement: Supplementary file 1 [file Table1.docx]

**SUPPLEMENTARY APPENDIX**

**
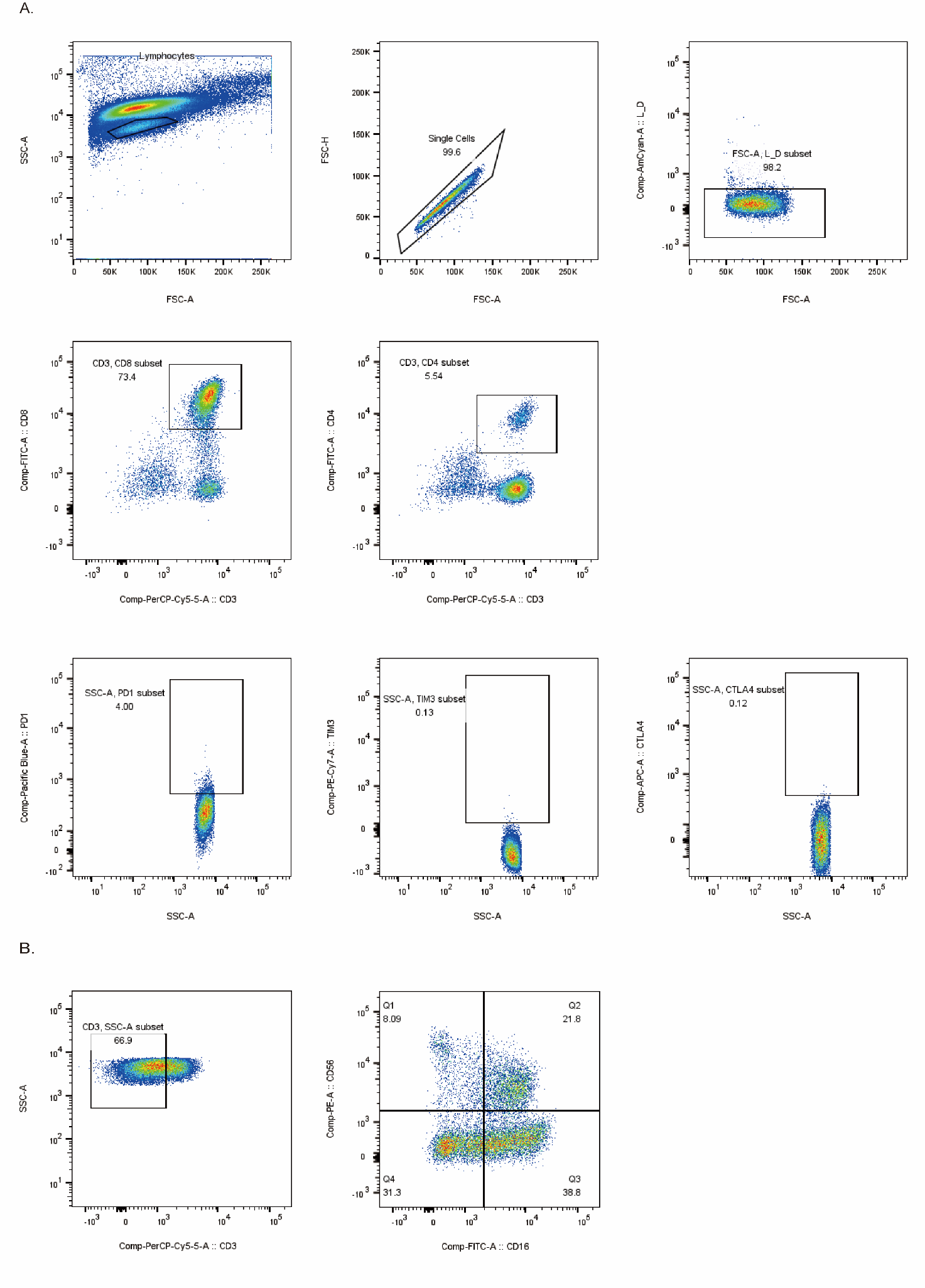
**

**Figure S1. Flow Cytometry gate strategy diagram for** **immune checkpoint molecule expression analysis.**

Lymphocyte populations were first identified through forward scatter (FSC) and side scatter (SSC) plots, and dead cells/debris and doublets were removed as indicated by the FSC Area /live-dead (L-D) viability dye and FSC Area/FSC Height plots. CD3-positive cells were then selected and further subdivided into CD8 and CD4 subsets, followed by an analysis of immune checkpoint molecule such as PD1, TIM3, and CTLA-4.

**
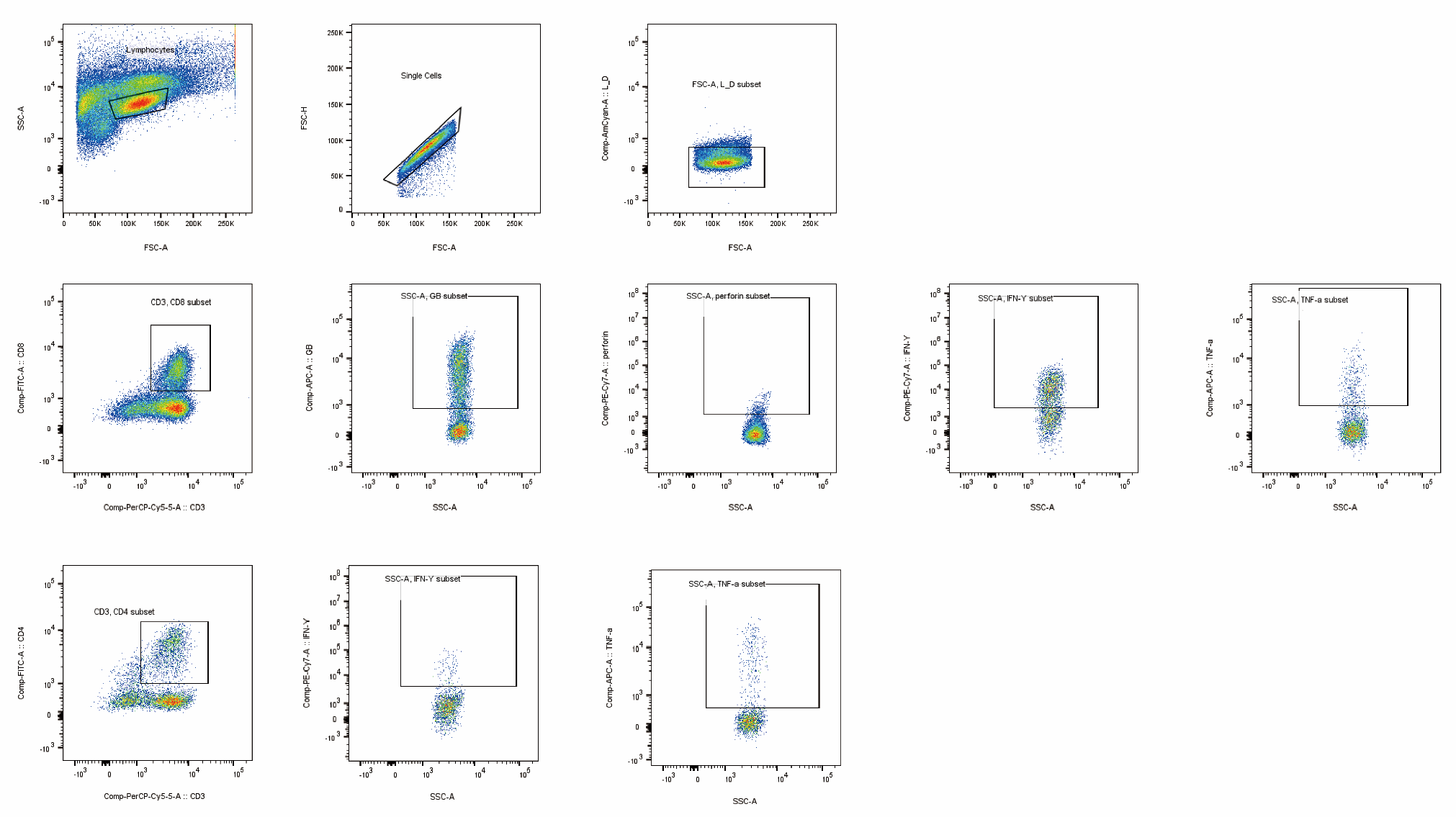
**

**Figure S2. Flow Cytometry gate strategy diagram for** **T cell cytotoxic function analysis.**

Lymphocyte populations were first identified through forward scatter (FSC) and side scatter (SSC) plots, and dead cells/debris and doublets were removed as indicated by the FSC Area /live-dead (L-D) viability dye and FSC Area/FSC Height plots. CD3-positive T cells were further subdivided into CD8 and CD4 subsets, and functional markers such as Granzyme B, Perforin, IFN-γ, and TNF-α were assessed in these populations to understand their cytotoxic and immune response characteristics.


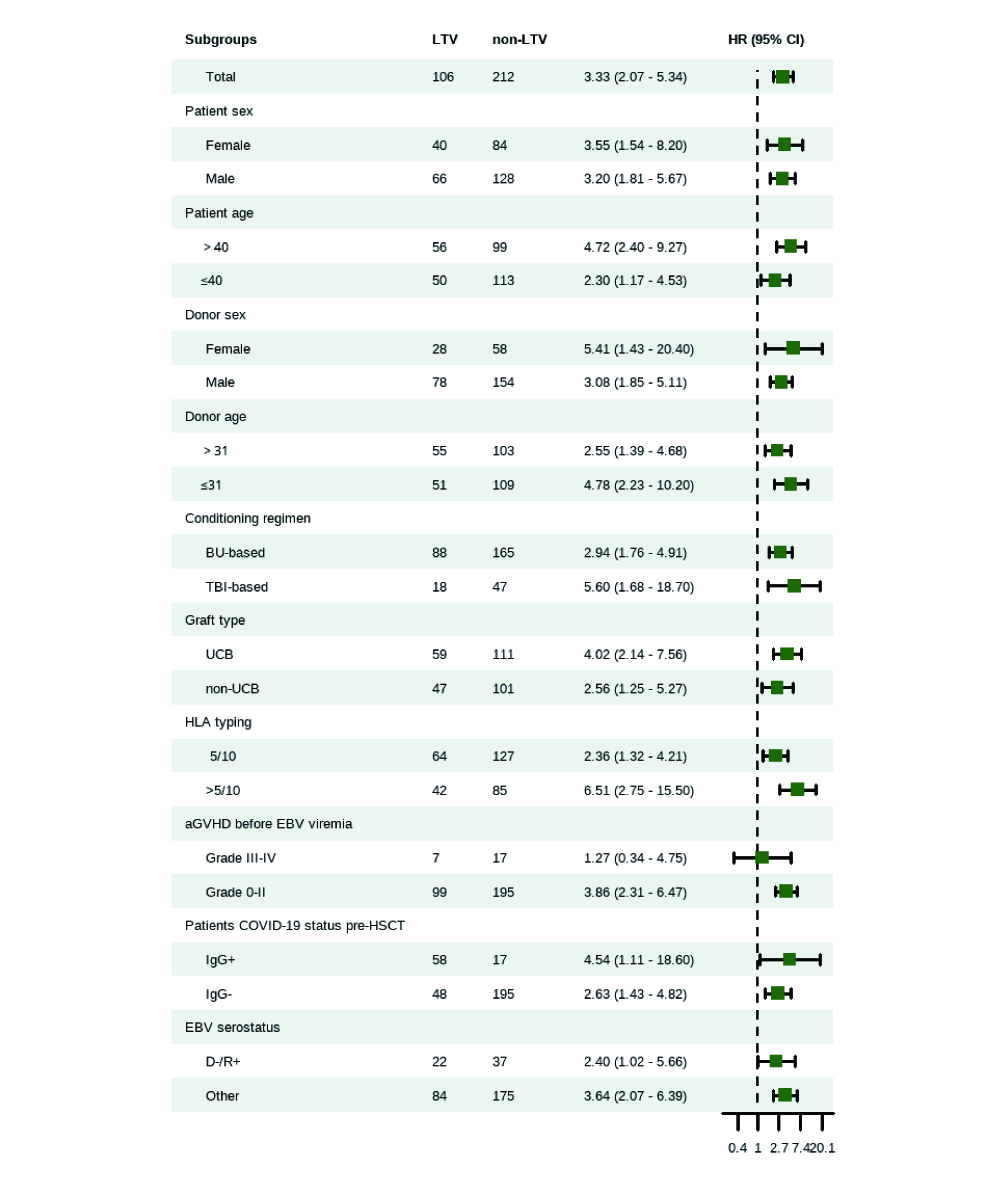


**Figure S3 Subgroup analyses of EBV viremia between LTV and non-LTV groups**

Abbreviations:  LTV, Letermovir; Bu, Busulfan; TBI, Total Body Irradiation; UCB, Umbilical Cord Blood; HLA, Human Leukocyte Antigen; aGVHD，Acute graft versus host disease; EBV, Epstein-Barr virus; HR, Hazard ratio.

**.**


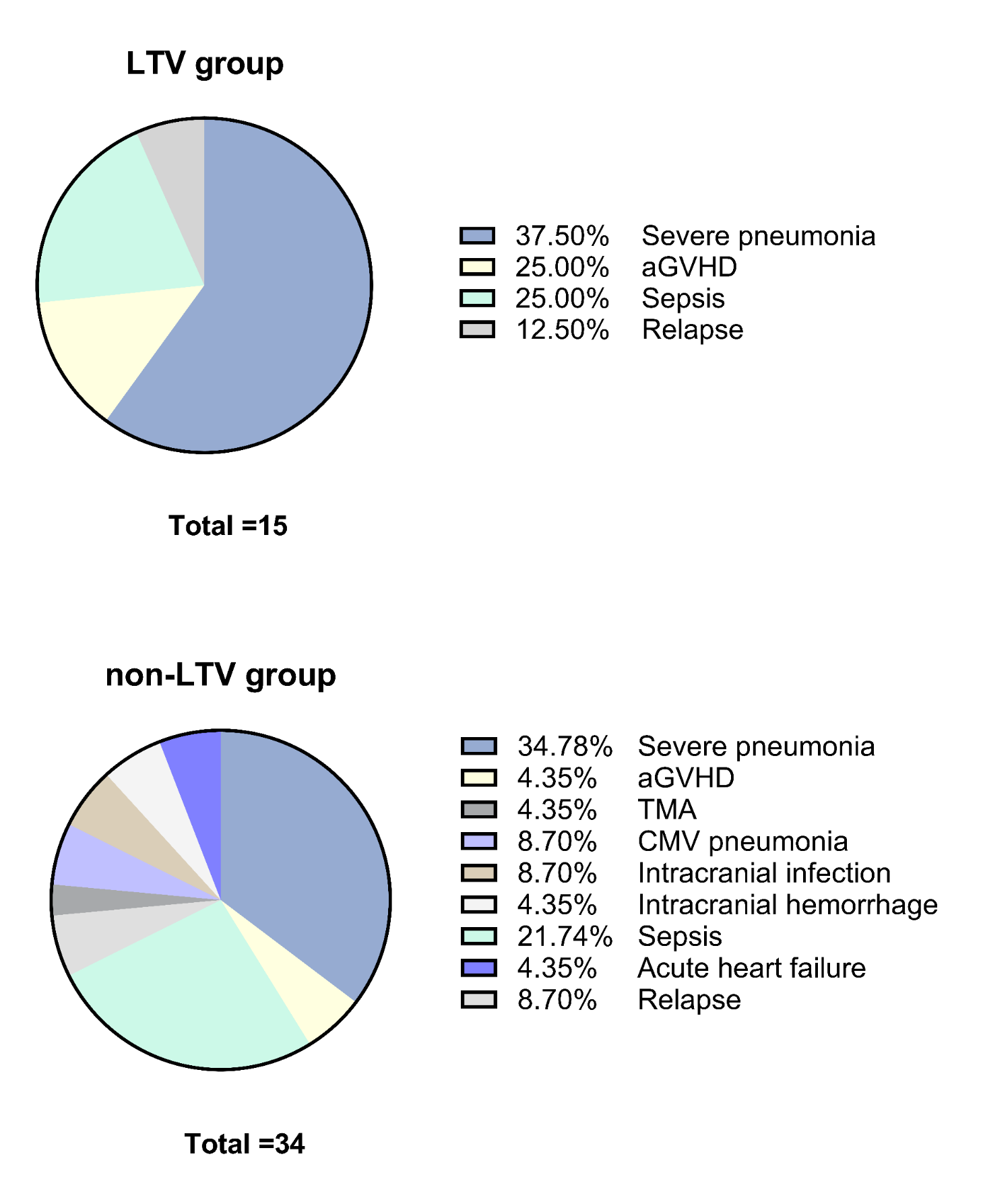


**Figure S4. Cause of death post-transplantation**

Abbreviations: LTV, Letermovir; aGVHD，Acute graft versus host disease; CMV, Cytomegalovirus; TMA, Thrombotic microangiopathy.


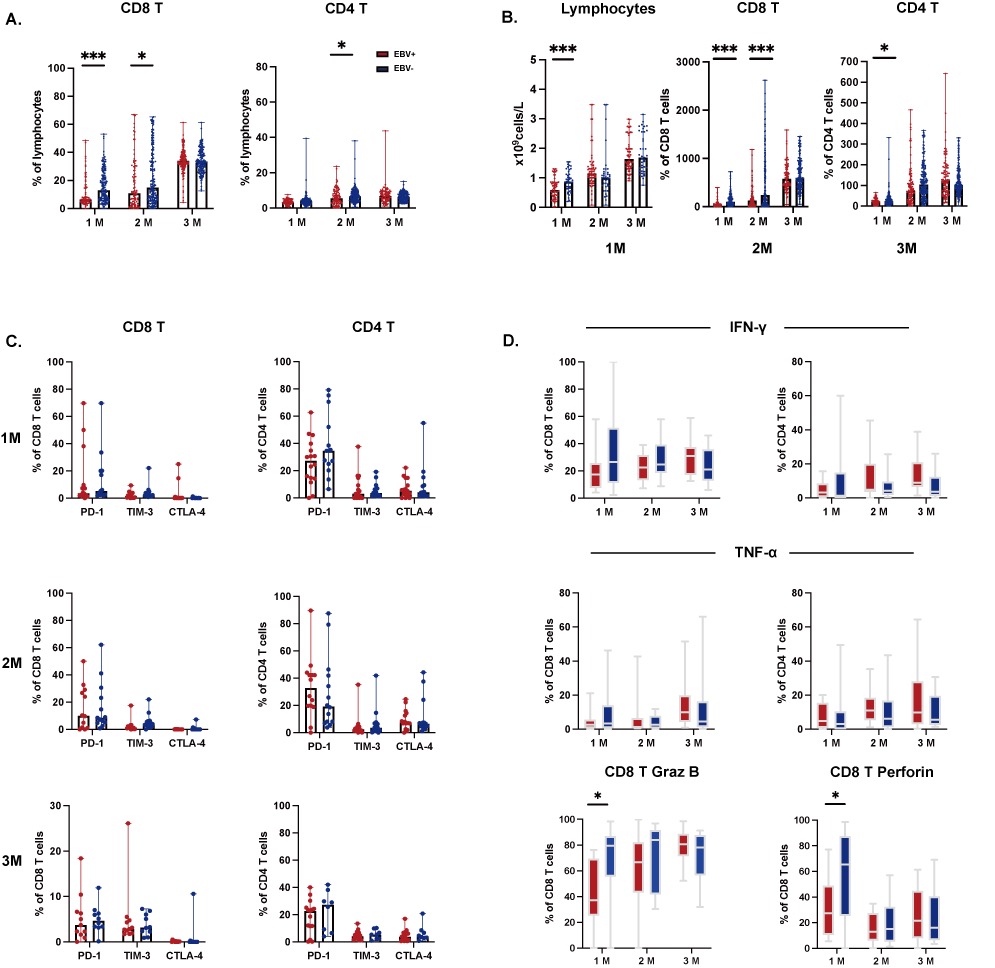


**Figure S5.** **Post-Transplant Lymphocyte Subsets and Functions: EBV+ vs. EBV- patients.** CD8 and CD4 T lymphocyte subsets percentages (**A**), CD8 and CD4 lymphocyte subsets counts (**B**), Expression of exhaustion markers on lymphocyte subsets (**C**), Function of lymphocyte subsets (**D**) within three months post-transplantation in the EBV+ and EBV- groups.

Abbreviations: EBV, Epstein-Barr virus; M, Month; *P < 0.05, ***P < 0.001.

**Table S1. Uni- and Multivariate Cox Proportional Hazard Regression Analyses for OS, DFS and GRFS.**

| **Risk factors** | **OS** | | **DFS** | | **GRFS** | |
| --- | --- | --- | --- | --- | --- | --- |
|  | **Univariate**  **P**  **(HR,95%CI)** | **Multivariate**  **P**  **(HR,95%CI)** | **Univariate**  **P**  **(HR,95%CI)** | **Multivariate**  **P**  **(HR,95%CI)** | **Univariate**  **P**  **(HR,95%CI)** | **Multivariate**  **P**  **(HR,95%CI)** |
| **Group** | | | | | | |
| LTV vs. non-LTV | 0.826  (0.93, 0.50-1.73) | 0.727  (0. 87, 0.41-1.88) | 0.439  (1.22, 0.74-2.03) | 0.638  (1.16, 0.63-2.14) | 0.759  (1.08, 0.67-1.72) | 0.747  (1.10, 0.63-1.92) |
| **Patient’s sex** | | | | | | |
| Female vs. male | 0.723  (1.11, 0.62-2.00) | 0.626  (1.18, 0.61-2.31) | 0.635  (1.13, 0.68-1.86) | 0.412  (1.27, 0.72-2.23) | 0.878  (0.96., 0.61-1.53) | 0.805  (1.07, 0.64-1.78) |
| **Patient’s age (Median)** | | | | | | |
| ＞40 vs.≤40 | 0.074  (1.71, 0.95-3.10) | 0.124  (1.73, 0.86-3.47) | 0.069  (1.59, 0.96-2.63) | 0.136  (1.56, 0.87-2.78) | 0.666  (1.10, 0.70-1.73) | 0.624  (1.14, 0.67-1.95) |
| **Conditioning regimen** | | | | | | |
| BU-based vs. TBI-based | 0.841  (0.93, 0.46-1.88) | 0.822  (0.91, 0.39-2.12) | 0.705  (0.89, 0.49-1.62) | 0.770  (0.90, 0.44 -1.84) | 0. 381  (0.79, 0.47-1.34) | 0.406  (0.77, 0.41-1.44) |
| **Graft type** | | | | | | |
| UCB vs. non-UCB | 0.790  (0.93, 0.52-1.65) | 0.685  (0.87, 0.46-1.67) | 0.398  (0.81, 0.49-1.32) | 0.550  (0.85, 0.49-1.47) | 0. 641  (0.90, 0.57-1.41) | 0.764  (1.08, 0.65-1.79) |
| **Disease status** | | | | | | |
| CR vs. non-CR | 0.840  (1.10, 0.43-2.78) | 0.222  (2.12, 0.63-7.10) | 0.822  (1.09, 0.50-2.40) | 0.142  (2.18, 0.77-6.17) | 0.684  (1.16, 0.56-2.42) | 0.281  (1.67, 0.66-4.24) |
| **Cytogenetics** | | | | | | |
| High-risk vs. low/ Intermediate-risk | **0.006**  (2.30, 1.27-4.17) | 0.583  (1.25, 0.57-2.74) | 0.674  (0.86, 0.42-1.76) | 0.636  (0.84, 0.40-1.76) | 0.967  (1.01, 0.54-1.90) | 0.889  (0.96, 0.50-1.83) |
| **aGVHD** | | | | | | |
| Grade III-IV vs. Grade 0-II | **0.004**  (3.08, 1.44-6.61) | **0.013**  (3.01, 1.26-7.20) | **0.002**  (2.85,1.45-5.60) | **0.007**  (2.90, 1.34-6.27) | - | - |
| **CMV viremia** |  |  |  |  |  |  |
| Y vs. N | 0.830  (0.93, 0.50-1.73) | 0.067  (1.89, 0.96-3.73) | 0.110  (1.50., 0.91-2.46) | 0.264  (1.38, 0.78-2.43) | **0.044**  (1.59, 1.01-2.49) | 0.132  (1.49, 0.89-2.49) |
| **EBV viremia** |  |  |  |  |  |  |
| Y vs. N | **0.011**  (2.17, 1.19-3.95) | 0.091  (1.91, 0.90-4.02) | **0.003**  (2.16, 1.29-3.60) | 0.064  (1.80, 0.97-3.35) | **0.005**  (1.97, 1.23-3.16) | 0.087  (1.65, 0.93-2.92) |
| **CMV serostatus** |  |  |  |  |  |  |
| D+/R+ vs. D−/R+ | 0.350  (0.62, 0.22-1.72) | 0.305  (0.57, 0.20-1.66/) | 0.910  (0.96, 0.46-2.01) | 0.787  (0.90, 0.41-1.96/) | 0.400  (1.31, 0.71-2.42) | 0.263  (1.45, 0.76-2.76/) |

Abbreviations: HR, Hazard ratio; LTV, Letermovir; BU, Busulfan; TBI, Total Body Irradiation; UCB, Umbilical Cord Blood; CR, Complete Remission; aGVHD，acute graft versus host disease; CMV, Cytomegalovirus; EBV, Epstein-barr virus; Y, Yes; N, No; D, Donor; R, Recipient

**Table S2.** **Patients’ lymphocyte subsets and functions within three months post-transplantation.**

|  | LTV | non-LTV | P |
| --- | --- | --- | --- |
| CD8 T, % [Median, (Min, Max)] | | | |
| +1M | 5.60% (0.25–47.00) | 16.90% (0.11–53.00) | < 0.001 |
| +2M | 7.40% (0.01–54.60) | 19.50% (0.80–66.90) | <0.001 |
| +3M | 32.45% (4.33–52.40) | 33.40% (12.50–61.30) | 0.060 |
| CD4 T, % [Median, (Min, Max)] | | | |
| +1M | 3.47 (0.65, 39.40) | 3.87 (0.05, 15.90) | 0.183 |
| +2M | 6.63 (0.07, 38.10) | 6.42 (0.56, 12.99) | 0.948 |
| +3M | 6.81 (1.12, 43.70) | 6.51 (2.44, 11.04) | 0.590 |
| CD56posCD16dim/neg, % [Median, (Min, Max)] | | | |
| +1M | 0.85 (0.00, 22.60) | 6.30 (0.01, 22.70) | 0.325 |
| +2M | 3.45 (0.05, 22.70) | 6.45 (1.05, 21.70) | 0.075 |
| +3M | 4.05 (0.28, 13.20) | 6.49 (0.95, 30.40) | 1.000 |
| CD56posCD16pos, % [Median, (Min, Max)] | | | |
| +1M | 0.09 (0.00, 22.70) | 0.08 (0.00, 19.30) | 0.984 |
| +2M | 0.79 (0.00, 36.40) | 8.81 (0.00, 29.30) | 0.051 |
| +3M | 1.24 (0.05, 20.50) | 2.67 (0.01, 14.50) | 0.876 |
| CD56dimCD16pos, % [Median, (Min, Max)] | | | |
| +1M | 24.70 (1.55, 82.20) | 17.60 (0.40, 82.20) | 0.370 |
| +2M | 19.65 (6.82, 72.18) | 20.60 (2.20, 51.40) | 0.727 |
| +3M | 10.20 (0.31, 55.80) | 11.50 (0.20, 47.10) | 0.530 |
| Lymphocytes, x10^9^ cells/L [Median, (Min, Max)] | | | |
| +1M | 0.72 (0.02, 1.54) | 0.72 (0.01, 4.55) | 0.610 |
| +2M | 1.08 (0.08, 3.48) | 1.75 (0.14, 4.33) | <0.001 |
| +3M | 1.71 (0.75, 3.15) | 1.85 (0.38, 3.75) | 0.084 |
| CD8 T, cells/uL [Median, (Min, Max)] | | | |
| +1M | 39.38 (0.76, 723.80) | 93.06 (0.28, 636.00) | < 0.001 |
| +2M | 77.39 (0.03, 1183.20) | 284.40 (8.06, 2619.65) | <0.001 |
| +3M | 554.86 (42.38, 1359.04) | 606.43 (99.18, 1587.60) | 0.056 |
| CD4 T, cells/uL [Median, (Min, Max)] | | | |
| +1M | 22.69 (0.20, 330.96) | 23.73 (0.18, 227.05) | 0.740 |
| +2M | 68.49 (0.50, 466.32) | 111.87 (3.14, 396.89) | <0.001 |
| +3M | 119.20 (15.34, 642.39) | 105.53 (19.23, 330.82) | 0.937 |
| CD8 PD-1 T/ CD8 T, % [Median, (Min, Max)] | | |  |
| +1M | 5.21 (1.12, 69.70) | 3.13 (0.00, 69.70) | 0.984 |
| +2M | 9.71 (0.00, 62.10) | 8.74 (0.82, 30.60) | 0.567 |
| +3M | 3.39 (0.04, 10.40) | 6.29 (0.90, 18.40) | 0.058 |
| CD8 TIM-3 T/ CD8 T, % [Median, (Min, Max)] | | |  |
| +1M | 1.95 (0.29, 9.37) | 2.19 (0.00, 22.10) | 0.647 |
| +2M | 2.56 (0.00, 17.50) | 2.86 (1.13, 22.00) | 0.338 |
| +3M | 3.20 (0.98, 26.10) | 2.84 (0.92, 5.52) | 0.310 |
| CD8 CTLA-4 T/ CD8 T, % [Median, (Min, Max)] | | |  |
| +1M | 0.19 (0.00, 14.60) | 0.91 (0.00, 25.00) | 0.303 |
| +2M | 0.10 (0.00, 7.32) | 0.03 (0.00, 0.15) | 0.261 |
| +3M | 0.09 (0.00, 10.60) | 0.02 (0.00, 0.19) | 0.095 |
| CD4 PD-1 T/ CD4 T, % [Median, (Min, Max)] | | | |
| +1M | 27.60 (0.00, 75.30) | 32.70 (6.15, 79.30) | 0.394 |
| +2M | 25.80 (0.00, 87.50) | 19.7 (3.60, 89.60) | 0.677 |
| +3M | 15.40 (0.33, 30.30) | 25.20 (00.33, 42.20) | 0.118 |
| CD4 TIM-3 T/ CD4 T, % [Median, (Min, Max)] | | | |
| +1M | 3.19 (0.00, 16.30) | 3.51 (0.00, 37.70) | 0.913 |
| +2M | 2.73 (0.00, 41.90) | 3.67(1.17, 14.50) | 1.000 |
| +3M | 4.72 (1.07, 13.50) | 4.77 (1.31, 9.92) | 0.740 |
| CD4 CTLA-4 T/ CD4 T, % [Median, (Min, Max)] | | | |
| +1M | 3.19 (0.00, 16.30) | 6.15 (0.00, 55.00) | 0.195 |
| +2M | 6.98 (0.00, 44.30) | 6.82 (1.06, 24.10) | 0.517 |
| +3M | 4.04 (0.69, 20.90) | 3.97 (0.62, 8.64) | 0.487 |
| CD8 Granzyme B+ T/ CD8 T, % [Median, (Min, Max)] | | | |
| +1M | 52.05 (0.00, 87.20) | 72.45 (11.10, 98.40) | 0.223 |
| +2M | 52.50 (0.00, 85.00) | 83.10 (37.20, 99.90) | 0.014 |
| +3M | 80.55 (51.60, 92.80) | 81.20 (31.90, 98.50) | 0.860 |
| CD8 Perforin T/ CD8 T, % [Median, (Min, Max)] | | | |
| +1M | 65.45 (0.00, 91.20) | 27.45 (5.56, 98.50) | 0.100 |
| +2M | 10.20 (0.00, 34.50) | 27.55 (1.27, 57.00) | 0.040 |
| +3M | 20.00 (0.33, 59.90) | 18.10 (0.68, 69.00) | 0.781 |
| CD8 TNF-α T/ CD8 T, % [Median, (Min, Max)] | | | |
| +1M | 2.95 (0.00, 42.90) | 3.99 (0.00, 46.20) | 0.664 |
| +2M | 1.48 (0.00, 11.80) | 1.52 (0.03, 42.70) | 0.865 |
| +3M | 9.35 (0.66, 51.50) | 6.20 (0.27, 66.00) | 0.980 |
| CD8 IFN-γ T/ CD8 T, % [Median, (Min, Max)] | | | |
| +1M | 16.35 (2.27, 100.00) | 32.4 (8.55, 100.00) | 0.110 |
| +2M | 29.80 (8.82, 57.90) | 19.80 (7.21, 47.10) | 0. 252 |
| +3M | 32.25 (6.17, 58.90) | 21.05 (6.03, 46.00) | 0.274 |
| CD4 TNF-α T/ CD4 T, % [Median, (Min, Max)] | | | |
| +1M | 3.60 (0.00, 90.90) | 5.00 (0.00, 49.40) | 0.285 |
| +2M | 8.75 (0.00, 35.60) | 10.26 (0.04, 43.40) | 4.443 |
| +3M | 9.33 (1.53, 64.40) | 9.61 (0.30, 36.10) | 0.705 |
| CD4 IFN-γ T/ CD4 T, % [Median, (Min, Max)] | | | |
| +1M | 2.40 (0.00, 90.90) | 2.62 (0.00, 60.00) | 0.853 |
| +2M | 3.66 (0.00, 15.30) | 6.23 (0.11, 45.40) | 0.178 |
| +3M | 9.95 (0.19, 38.80) | 6.42 (0.09, 26.00) | 0.347 |

Abbreviations: LTV, Letermovir; M, Month.
